# Population pharmacokinetics and pharmacodynamics of cysteamine in nephropathic cystinosis patients

**DOI:** 10.1186/1750-1172-6-86

**Published:** 2011-12-23

**Authors:** Naïm Bouazza, Jean-Marc Tréluyer, Chris Ottolenghi, Saik Urien, Georges Deschenes, Daniel Ricquier, Patrick Niaudet, Bernadette Chadefaux-Vekemans

**Affiliations:** 1 EA 3620, Université Paris Descartes, Sorbonne Paris Cité, France; 2 Unité de Recherche clinique, AP-HP, Hôpital Tarnier, Paris, France; 3 Service de Pharmacologie Clinique, AP-HP, Hôpital Cochin-Saint-Vincent-de-Paul, France; 4 CIC-0901 Inserm, Cochin-Necker, Paris, France; 5 Unité de Biochimie Métabolique, Hôpital Necker-Enfants Malades, Paris, France; 6 Service de Néphrologie Pédiatrique, Hôpital Necker-Enfants Malades, Paris, France; 7 Unité de Néphrologie Pédiatrique, Hôpital Robert Debré, Paris, France

**Keywords:** population pharmacokinetics, pharmacodynamics, cysteamine, cystinosis

## Abstract

**Background:**

Nephropathic cystinosis is an autosomal recessive disorder resulting in an impaired transport of cystine trough the lysosomal membrane causing an accumulation of free cystine in lysosomes. The only specific treatment for nephropathic cystinosis is cysteamine bitartrate. This study was aimed to describe the relationship between cysteamine plasma concentrations and white blood cell cystine levels, and to simulate an optimized administration scheme to improve the management of patients with cystinosis.

**Methods:**

Cysteamine and cystine concentrations were measured in 69 nephropathic cystinosis patients. A total of 250 cysteamine plasma concentrations and 243 intracellular cystine concentrations were used to perform a population pharmacokinetic and pharmacodynamic analysis. An optimized administration scheme was simulated in order to maintain cystine levels below 1 nmol half-cystine/mg of protein and to investigate the possibility of administrating the treatment less than 4 times a day (QID, recommended). The current dosing recommendations are 1.3 g/m^2^/day for less than 50 kg BW and 2 g/day thereafter; the maximum dose should not exceed 1.95 g/m^2^/day.

**Results:**

Cysteamine concentrations were satisfactorily described by a one-compartment model. Parameter estimates were standardized for a mean standard bodyweight using an allometric model. WBC cystine levels were adequately described by an indirect response model where the first-order removal rate constant is stimulated by the cysteamine concentrations.

**Conclusions:**

According to simulations, in order to increase the percentage of patient with cystine levels below 1 nmol half-cystine/mg of protein, the current dosages could be changed as follows: 80 mg/kg/day (QID) from 10 to 17 kg, 70 mg/kg/day (QID) from 17 to 25 kg, 60 mg/kg/day (QID) from 25 to 40 kg and 50 mg/kg/day (QID) from 40 to 70 kg (these dosages remain under the maximum recommended dose). However an 8-hourly daily treatment (TID) did not provide acceptable cystine levels and should not be proposed.

## Background

Nephropathic cystinosis is an autosomal recessive disorder resulting in an impaired transport of cystine trough the lysosomal membrane causing an accumulation of free cystine in lysosomes [1]. Lysosomal cystine accumulation leads to the formation of intracellular crystal throughout the body. The kidney is the most damaged organ, leading to progressive renal failure. Without specific therapy, end stage renal failure occurs before 10 years of age [2]. Patients with cystinosis suffer additionally from severe growth retardation and photophobia due to the accumulation of cystine crystals in the cornea [1].

The only specific treatment for nephropathic cystinosis is cysteamine bitartrate (Cystagon^®^) which has changed the course of the disease. Treatment reduces the progression of renal impairment and is more effective when initiated before 2 years old when renal function is still normal [3]. It is recommended to continue cysteamine treatment in patient with nephropathic cystinosis after renal transplantation to limit cystine accumulation in extra-renal tissues.

Limited studies are available on the pharmacokinetics and pharmacodynamics of cysteamine, most of them involving healthy adult subjects or a very small number of pediatric patients [4-6]. Cysteamine bitartrate is usually administered orally every 6 hours. The current recommendations are 1.3 g/m^2^/day for children less than 50 kg, and 2 g/day thereafter. The dose of cysteamine should not exceed 1.95 g/m^2^/day, the maximum dose used in clinical trials [3].

In the present study, we have developed a population pharmacokinetic model for cysteamine bitartrate in a large group of nephropathic cystinosis patients from children to adult in order to determine the relationship between cysteamine plasma concentration and white blood cell (WBC) cystine levels. Using this model, an optimized administration scheme is suggested to maintain cystine levels below 1 nmol half-cystine/mg of protein.

## Methods

### Patients and treatment

The population comprises 69 nephropathic cystinosis patients, ranging in age from 0.4 to 36 years (mean 12.5 years), and in bodyweight from 7.6 to 83 kg (mean 34.3 kg). Patients received cysteamine bitartrate as capsule every 6 hours for the treatment of nephropathic cystinosis, the mean (standard deviation) cysteamine dose was 35.5 (21) mg.kg^-1^/day.

### Study design

13 cystinotic patients ranging in age from 2.5 to 28 years and in bodyweight from 13.7 to 80.2 kg on cysteamine bitartrate were involved in an open-label study, at steady state. The study consisted of collecting blood samples from non-transplant patients receiving cysteamine before dose and 2, 3, 4, and 6 h after administration. These samples were used for plasma cysteamine concentration assay and for WBC cystine concentration assays.

On the other hand, 56 patients ranging in age from 0.4 to 36 years and in bodyweight from 7.6 to 83 kg were involved in a monitoring routine basis.

### Analytical method

#### Sample collection

Venous blood samples were collected into citric acid-citrate-dextrose (ACD) tubes. For the monitoring of treatment with cysteamine, samples were collected 6 hours post-dose. Whole blood was conserved at room temperature until the sample was prepared. The plasma was separated from the leucocytes and erythrocytes by centrifugation at 1200 g for 15 min. The plasma layer was collected and stored at -20°C until analysis of cysteamine. The middle fraction containing total leucocytes was collected and PMN cells were isolated according to guidelines from the group "Cystine in white blood cells" [7].

#### Determination of cysteamine

Cysteamine was determined as total cysteamine, by liquid chromatography-MS/MS.

Sample preparation was carried out by adding 10 μl of internal standard (d4-deutered cysteamine) 100 μM, 10 μL of DTT 0.1 M for reduction of disulfide bond to 200 μl of plasma. After incubation at 37°C for 15 min, 30 μL of 12% sulfosalicylic acid was added to deproteinize the sample. After centrifugation, 20 μl of 5.2 mM N-ethylmaleimide (NEM) was added. 5 μL was then injected into LC-MS/MS. Chromatographic separation was achieved on an Agilent 1100 system (Agilent Technologies, Waldbronn, Germany) using a XD8-C8 column (150 × 4.6 mm, reference UP3OD#3QS; Interchim). The column was eluted at 600 μl/min using an isocratic mobile phase of water/acetonitrile (410/90) with 0.1% formic acid.

The column eluate was injected directly into the Applied Biosystems/MDS Sciex API3000 MSMS which was maintained in an electrospray positive mode.

#### Determination of cystine

PMN cells cystine levels, reported as nmol half-cystine/mg protein were measured using liquid chromatography-tandem mass spectroscopy (API 3000LC/MS/MS; Applied Biosystems/MDS Sciex) with previously described methods [8].

### Modeling strategy and population pharmacokinetic-pharmacodynamic model

Data were analysed using the nonlinear mixed effect modelling software program Monolix version 31s http://wfn.software.monolix.org[9]. Parameters were estimated by computing the maximum likelihood estimator of the parameters without any approximation of the model (no linearization) using the stochastic approximation expectation maximization (SAEM) algorithm combined to a MCMC (Markov Chain) monte Carlo) procedure. The number of MCMC chains was fixed to 5 for all estimations. The between-subject variabilities (BSV or η) were ascribed to an exponential model. Parameter shrinkage was calculated as {1-sd(eta)/omega}, where sd(eta) and omega are the standard deviation of individual eta parameters and the population model estimate of the BSV respectively [10]. The Likelihood Ratio Test (LRT) including the log-likelihood, the Akaike information criterion (AIC) and the bayesian information criterion (BIC) were used to test different hypotheses regarding the final model, covariate effect(s) on pharmacokinetic parameter(s), residual variability model (proportional versus proportional plus additive error model), and structure of the variance-covariance matrix for the BSV parameters.

Main covariates of interest in the population were age, bodyweight (BW), creatinine clearance, size, and body surface area (BSA).

Pharmacokinetics parameter estimates were standardized for a mean standard bodyweight using an allometric model: P_i _= P_STD _× (BW_i_/BW_STD_)^PWR^

Where P_STD _is the standard value of parameter for a patient with the standard bodyweight value and P_i _and BW_i _are the parameter and bodyweight of the ith individual. The PWR exponents may be estimated from the data. However, from allometric scaling theory these are typically 0.75 for clearance parameters and 1 for volumes of distribution [11].

For evaluation of the goodness-of-fit, the following graphs were performed for the final model: observed and predicted concentrations versus time, observed concentrations vs population predictions, weighted residuals vs time and weighted residuals vs predictions. Similar graphs using individual predictive estimation were examined. Diagnostic graphics were obtained using the R program [12].

The pharmacokinetic parameters were then fixed and the parameters of the pharmacodynamic model were estimated. An indirect response model with a zero-order production rate constant (K_in_) and a first-order removal rate constant (K_out_) was used to describe the WBC cystine levels. Cysteamine stimulates the first-order removal rate constant to allow the cystine depletion according to the following equation:

$$\frac{\text{dR}}{\text{dt}}=\;{\text{K}}_{\text{in}}-{\text{K}}_{\text{out}}\;\text{. }\left(\frac{{\text{E}}_{\text{max}}\;\text{. }{\text{C}}_{\text{P}}}{\text{E}{\text{C}}_{\text{50}}\text{ + }{\text{C}}_{\text{P}}}\right)\;\times \text{ R}$$

where R is the pharmacodynamic response, E_max _is the maximum effect, C_p _is the cysteamine concentration, EC_50 _is the concentration producing 50% of maximal response. K_in _is derived from K_out _and Ro, baseline, as K_in _= K_out _× Ro. A simultaneous pharmacokinetic-pharmacodynamic modeling was then performed to estimate both pharmacokinetic and pharmacodynamic parameters.

### Visual predictive check (VPC) validation

Cysteamine and cystine concentration profiles were simulated and compared with the observed data to evaluate the predictive performance of the model. Simulated concentrations were then compared with the observed data to evaluate the predictive performance of the model. The vector of pharmacokinetic parameters was simulated using the final model. Each vector parameter was drawn in a log-normal distribution with a variance corresponding to the BSV previously estimated. A simulated residual error was added to each simulated concentration. All observed and simulated concentrations were standardized for a cysteamine bitartrate dose of 600 mg/day. The 5^th^, 50^th ^and 95^th ^percentiles of the simulated concentrations at each time were then overlaid on the observed concentration data and a visual inspection was performed. The variability was reasonably estimated if the 95% confidence interval for the proportion of observed data outside the bounds included the theoretical value of 10%.

### Doses simulations

To propose doses in mg/kg not exceeding the maximum dose of 1.95 g/m^2^/day, the following formula for the pediatric population: BSA = [(4 × BW) + 7]/(BW + 90) (Additional information can be found at the "Société Francophone de Médecine d'Urgence", website http://www.sfmu.org/fr/formation/calculateurs#Pediatrie) was used to represent the maximum cysteamine dose in mg/kg as a function of bodyweight. Different doses per kilogram per day in function of bodyweight were then derived. The current recommendations as well as our derived daily doses were simulated for a TID and for a QID administration; the percentage of patient achieving WBC cystine levels below 1 or below 2 nmol half-cystine/mg of protein was then calculated and the cystine levels associated were drawn.

## Results

### Demographic data

250 cysteamine plasma concentrations and 243 intracellular cystine concentrations from 69 patients were available for pharmacokinetic and pharmacodynamic evaluation. The median cysteamine bitartrate daily dose administered 4 times a day was 1 g ranging from 200 mg to 2.7 g. Table 1 summarizes patients' characteristics.

**Table 1:** Characteristics of the nephropathic cystinosis patients (n = 69) enrolled in the pharmacokinetics and pharmacodynamics study.

	Mean	Min-Max
**Age (years)**	12.5	0.4 - 36
**Weight (kg)**	34.3	7.6 - 83
**Creatinine levels (μmol/L)**	125.2	24 - 800
**Creatine clearance (ml/min)**	88.6	6.4 - 172.5
**Taille (cm)**	127.4	67 - 177

### Cysteamine pharmacokinetics

**Figure 1:**
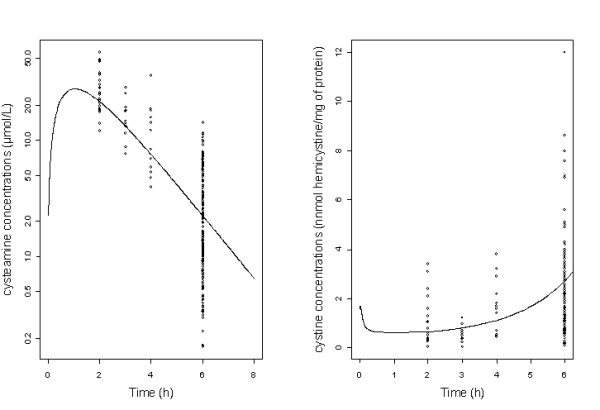
(A) Observed cysteamine concentrations (o) and population predicted cysteamine concentrations (curve) as a function of time in log scale; (B) Observed WBC cystine levels (o) and population predicted cystine concentrations (curve) as a function of time.

A one-compartment model adequately described the data, thus the apparent parameters of the model were the clearance (CL/F), the volume of distribution (V/F), and the absorption rate constant (Ka), F is the unknown bioavailability. Residual variability was best described by a proportional error model. Inter-subject variability was described by exponential error model and retained only for apparent clearance. The allometric scaling of clearance (CL/F) and volume term (V/F) improved the goodness of fit. None of the other covariates (age, creatinine clearance, size, BSA) had a significant effect on the model. Figure 1(A) displays cysteamine observed and predicted plasma concentrations as a function of time.

Table 2 summarizes the final population pharmacokinetic estimates. All the parameters were well estimated, given their relative standard error (RSE%). The η-shrinkage for CL/F was 0.13 indicating that the empirical Bayesian estimates for individual clearance parameter are reliable.

**Table 2:** Population pharmacokinetic and pharmacodynamic parameters of WBC cystine levels and cysteamine standardized for a weight of 70 kg.

Parameters	Mean	RSE (%)
*Structural pharmacokinetic model*		
**ka **(*h^-1^*)	1.38	7
**V/F **(*L/70 kg*)	82.4	11
**CL/F **(*L/h/70 kg*)	42.3	8
*Statistical pharmacokinetics model*		
**ω_CL/F_**	0.28	10
**σ**	0.45	5
		
*Structural pharmacodynamic model*		
**Ro **(*nmol half-cystine/mg of protein)*	1.59	9
**K_out _**(*h^-1^*)	20.2	33
**E_max_**	3.82	7
**EC_50 _***(μmol/L)*	12.5	15
*Statistical pharmacodynamic model*		
**ω_Ro_**	0.62	11
**σ**	0.49	5

*Key*: RSE%, relative standard error (standard error of estimate/estimate*100), Ka absorption rate constant, CL/F apparent elimination clearance, V/F apparent volume of distribution, Ro baseline (predose) cystine level, K_out _first-order removal rate constant, Emax maximum stimulation of K_out_, EC_50 _concentration resulting in 50% of maximal response, σ residual variability estimates, ω interindividual variability estimates. The typical parameters refers to an adult patient weighing 70 kg according to an allometric model: [Typical value] = [Typical parameter] * (bodyweight/70)^PWR ^where PWR = 0.75 for CL and 1 for V.

### WBC cystine levels

An indirect response model where the first-order removal rate constant (kout) is stimulated by the cysteamine concentrations adequately described the data. Residual variability was best described by a proportional error model. The inter-subject variability was described by exponential error model and retained only for baseline (predose) cystine level (Ro). Figure 1(B) displays WBC cystine levels observed and predicted concentrations as a function of time for the patients. Table 2 summarizes the final population pharmacodynamic estimates. All the parameters were well estimated, given their relative standard error (RSE%). The η-shrinkage for Ro was 0.15 indicating that the empirical Bayesian estimates for individual baseline parameter are reliable.

Figure 1(C) displays the cysteamine predicted plasma concentrations and WBC cystine levels predicted concentrations as a function of time for an administration of 600 mg/day.

### Evaluation and validation

**Figure 2:**
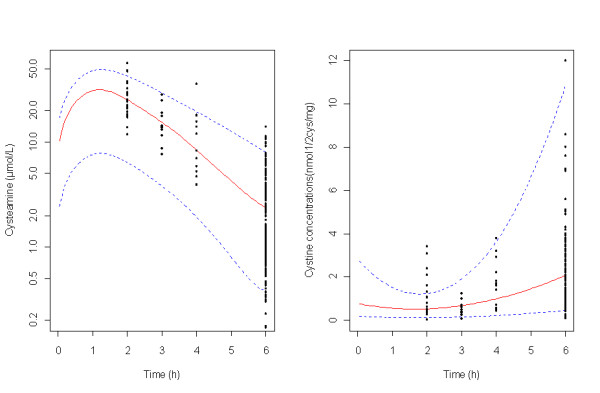
**Evaluation of the final model: comparison between the 5th, 50th and 95^th ^percentile obtained from 400 simulations (lines), and the observed data (o) for cysteamine concentrations standardized for a cysteamine dose of 900 mg/day (A) and for WBC cystine levels (B)**.

Figure 2 (VPC) shows that the average prediction matches the observed concentration time-courses and that the variability is reasonably estimated. The number (percentage) of observed points within the 90% prediction interval was for plasma cyteamine concentrations 232/250 (92.8%) and for WBC cystine levels 225/243 (92.5%).

### Doses simulations

**Figure 3:**
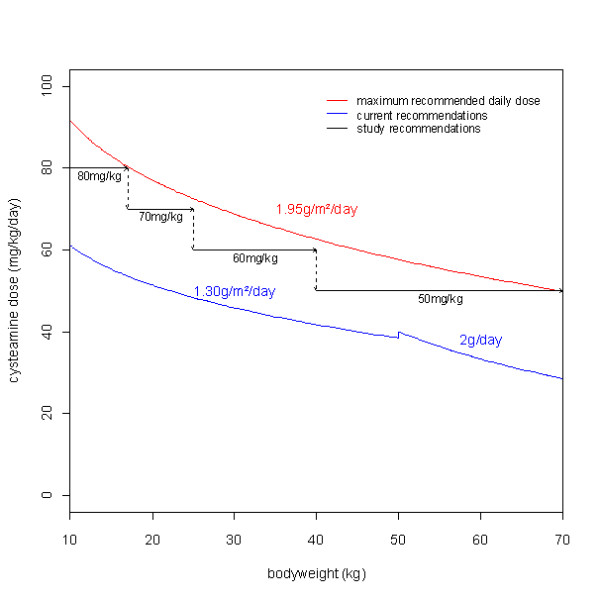
**Daily dose (mg/kg/day) of cysteamine as a function of bodyweight**. The red line corresponds to the maximum daily dose recommended. The blue line corresponds to the usual daily dose recommended. Black arrows correspond to study daily dose recommendations.

**Figure 4:**
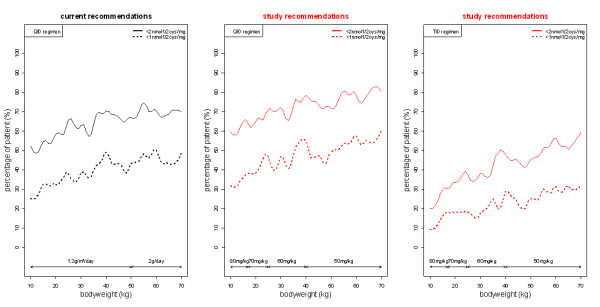
**Percentage of patients with cystine concentrations below 1 nmol half-cystine/mg of protein (dashed lines) or below 2 nmol half-cystine/mg of protein (lines) according to bodyweight for the usual recommendations and for our dosing scheme (mg/kg/day) administered either in QID regimen or TID regimen**.

Figure 3 shows the maximum cysteamine dose recommended and the usual recommendations as a function of bodyweight. Without exceeding the maximum recommended dose, increased doses could be provided as follows: 80 mg/kg/day from 10 to 17 kg, 70 mg/kg/day from 17 to 25 kg, 60 mg/kg/day from 25 to 40 kg and 50 mg/kg/day from 40 to 70 kg. Figure 4 displays the percentage of patient as a function of bodyweight with cystine levels below 1 and 2 nmol half-cystine/mg of protein with the usual recommendation, and with our proposed daily dose given 4 times a day or 3 times a day. Our daily dose proposition given 4 times a day led to a higher percentage of patient achieving satisfactory cystine levels from 10 to 70 kg.

However the percentage of patient achieving cystine levels below 1 and 2 nmol half-cystine/mg of protein with our daily dose proposal given 3 times a day remains very low and should not be proposed to patients.

**Figure 5:**
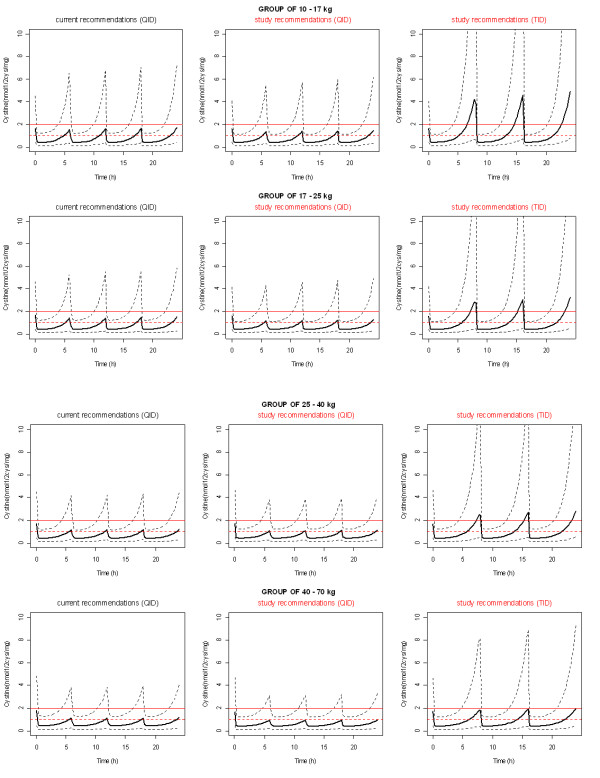
**Simulated cystine median (lines) and IC90 (dashed lines) concentrations (nmol half-cystine/mg of protein) according to bodyweight groups for the usual recommendations and for our dosing scheme (mg/kg/day) administrated either in QID regimen or TID regimen**.

The simulated profile of WBC cystine levels associated with the usual recommendation and with our proposed daily dose given 4 times a day or 3 times a day was then performed according to the bodyweight groups (10 to 17 kg, 17 to 25 kg, 25 to 40 kg, and 40 to 70 kg) defined by our dosing schedules (Figure 5). Our four daily doses appear to decrease in each group of bodyweight cystine levels compared to the usual recommendation. Nevertheless, our three daily doses did not provide satisfactory cystine levels.

Table 3 provides cysteamine residual concentrations according to bodyweight groups derived from the current recommendations and these study recommendations. With the current recommendations, the residual cysteamine concentrations of patients with satisfactory cystine levels were significantly higher in each bodyweight groups compared to patients with no satisfactory cystine levels (more than 2 nmol half-cystine/mg of protein). The residual cysteamine concentrations derived from this study recommendations were closed to those obtained in patients with low cystine levels treated with the current recommendations. Thus, cysteamine residual concentrations could be monitored instead of cystine levels (easier in practical terms) and propose our four daily dosing scheme instead of the current recommendations at least for patients with low values of residual cysteamine plasma concentration according to the corresponding bodyweight group (table 3).

**Table 3:** Residual cysteamine (6-hour post-dose) concentrations (μmoL/L) obtained with current recommendations and this study recommendations according to bodyweight groups.

	Residual cysteamine concentration (μmoL/L) median (sd)			
**WT**	**Current Recommendations**			**Study recommendations**

	*Patients with cystine > 2 *	*Patients with cystine < 2 *	*pvalue*	
10-17 kg	1.7 (2.8)	4.8 (5.7)	*< 10^-4^*	4.3 (7.0)
17-25 kg	2.1 (4.0)	6.1 (7.3)	*< 10^-4^*	5.4 (9.2)
25-40 kg	2.6 (3.9)	6.4 (7.6)	*< 10^-4^*	6.5 (9.4)
40-70 kg	3.3 (4.5)	7 (7.9)	*< 10^-4^*	7.9 (10.6)

## Discussion

Cystinosis is an inherited disorder characterized by defected lysosomal efflux of cystine causing a continuous accumulation of free cystine and intracellular crystal formation across the body. Without any treatment cystinosis progresses to renal failure. The therapeutic approach for cystinotic patients consists of cysteamine bitartrate which has been demonstrated to be effective in the depletion of intracellular cystine [1]. This paper describes the pharmacokinetics and the pharmacodynamics of cysteamine in 69 cystinotic patients aged 5 months to 36 years. Cysteamine concentrations were satisfactorily described by a one compartment model. In our model, no effect of age on clearance was observed following the bodyweight-based allometric scaling of the parameters. Creatinine clearance did not have an influence on the pharmacokinetic parameters which can be explained by the limited number of patients with severe renal insufficiency involved in the study; indeed, some data suggest that cysteamine pharmacokinetic parameters may not be significantly modified in patients with mild to moderate renal insufficiency [3]. One the other hand cysteamine is mainly eliminated via metabolism pathway.

To be effective, cysteamine has to pass through lysosomal membranes where it reacts with cystine to form a cysteine-cysteamine complex which can be depleted from the lysosome using the lysine transport system [13]. With respect to the mechanism of action of cysteamine, an indirect response model was used to describe the WBC cystine concentrations. Cystine was represented as an effect compartment with a zero-order production rate constant (K_in_) and a first-order removal rate constant (K_out_). To reproduce the cystine depletion caused by cysteamine a model where the drug concentration stimulates the exit constant (K_out_) was used. This model describes satisfactorily the link between cysteamine concentrations and WBC cystine levels. The therapeutic aim of the treatment of cystinosis is to maintain WBC cystine levels lower than 1 nmol half-cystine/mg of protein. At present, the rhythm of administration of cysteamine bitartrate is 4 times a day which is uncomfotable and may cause non-compliance. According to the model we simulated an optimized administration scheme which decreases the administration rhythm while maintaining cystine levels below 1 nmol half-cystine/mg of protein. It appears that the every 6-hourly daily treatment with cysteamine bitartrate could not be decreased to an 8-hourly daily treatment without exceeding the recommended maximum daily dose (1.95 g/m^2^/day used in clinical trials). However, the current recommended 6-hourly daily treatment could be improved by increasing the percentage of patient with satisfactory cystine levels (below 1 or 2 nmol half-cystine/mg of protein) which could be a way to improve the management of patients with cystinosis. Our modeling was based on data with 95% of the children weighing between 12 and 70 kg, thus to avoid risks of extrapolation from the model, our recommendation was based on a range from 10 to 70 kg.

## Conclusion

This study reports cysteamine pharmacokinetics and pharmacodynamics in nephropathic cystinosis patients. A model linking the cysteamine concentrations to the WBC cystine levels was performed. According to this model a dosing scheme not exceeding the maximum recommended daily dose was simulated to improve the cystine concentrations profile obtained with usual recommendation. According to the simulations, nephropathic cystinosis patients should receive the following cysteamine bitartrate dose: 80 mg/kg/day (QID) from 10 to 17 kg, 70 mg/kg/day (QID) from 17 to 25 kg, 60 mg/kg/day (QID) from 25 to 40 kg and 50 mg/kg/day (QID) from 40 to 70 kg. To limit the risk of drug toxicity, the starting dose of cysteamine should be 1/4 to 1/6 of the scheduled maintenance dose and progressively increased by 10 mg/kg increments every two weeks until the final dose is achieved [3]. However an 8-hourly daily treatment could not be proposed with regard to the maximum recommended daily dose of 1.95 g/m^2^/day.

## Competing interests

The authors declare that they have no competing interests.

## Authors' contributions

NB, CO, PN, BCV, DR and JMT designed research; NB, JMT, PN, BCV, DR and GD conducted research; NB, SU, and JMT analyzed data; NB and BCV wrote the paper; PN and JMT had primary responsibility for final content. All authors read and approved the final manuscript.
